# Laser Therapy Changes the Expression of Matrix Metalloproteinases in Bleomycin-Induced Skin Fibrosis

**DOI:** 10.3390/life13030810

**Published:** 2023-03-16

**Authors:** Anna G. Soboleva, Vladimir V. Sobolev, Mari M. Karapetyan, Alexandre Mezentsev, Olga I. Rud’ko, Evgenia D. Davydova, Julia A. Mogulevtseva, Olga V. Zhukova, Irina M. Korsunskaya

**Affiliations:** 1 Center for Theoretical Problems in Physico-Chemical Pharmacology, Russian Academy of Sciences, 30 Srednaya Kalitnikovskaya Street, 109029 Moscow, Russia; 2 Avtsyn Research Institute of Human Morphology of Petrovsky National Research Centre of Surgery, 3 Tsyurupy Street, 117418 Moscow, Russia; 3 LLC «Smile Zone», 41 Kolpakova Street, 141008 Mytishchi, Russia; 4 Faculty of Biology, M.V. Lomonosov Moscow State University, 1-12 Leninskie Gory, 119991 Moscow, Russia; 5 Department of Agronomy and Biotechnology, Russian Agrarian University (Moscow Timiryazev Agricultural Academy), 49 Timiryazeva Street, 127550 Moscow, Russia; 6 Moscow Center of Dermatology and Cosmetology, 17 Leninsky Avenue, 119071 Moscow, Russia

**Keywords:** cutaneous fibrosis, scars, matrix metalloproteinases, bleomycin-induced skin fibrosis, skin remodeling, wound healing, animal models, inflammation, immune response, skin damage

## Abstract

Matrix metalloproteinases (MMPs) are often considered biomarkers of skin fibrosis. At the early stages of the pathological process, an elevation of their enzymatic activity causes significant changes in the composition of the extracellular matrix. MMPs secreted by immune cells facilitate their migration to the site of damage. Then, the immune cells eliminate the affected cells and biomolecules. Moreover, bidirectional changes in the activity of proteolytic enzymes, including MMPs, accompany wound healing. This study aimed to assess changes in the expression of *Mmp2*, *Mmp3*, and *Mmp9* after treating mice with laser therapy using the experimental model of bleomycin-induced skin fibrosis. Using immunohistochemistry, we characterized the histological features of scarred skin. We also analyzed changes in the expression of MMPs using real-time polymerase chain reaction before and after laser irradiation. We showed that treatment of the mice with a CO_2_ laser partially normalized the histological features of scarred skin. We also noticed a decrease in the expression of *Mmp2*, *Mmp3* (both *p* < 0.05), and *Mmp9* (*p* = 0.065) during scar healing. The obtained results suggest that normalization of skin homeostasis requires control of MMP activity via induction of genes.

## 1. Introduction

Fibrosis is a pathological condition characterized by an expansion of connective tissue that compromises the integrity of the affected organ [1,2]. Trauma is a cause of cutaneous fibrosis. It may also appear at sites of burns or frostbite due to the developing inflammatory response. Scarring is one of the complications associated with skin fibrosis [3,4]. In the skin, the deposition of the extracellular matrix, primarily collagens, is an indispensable and reversible part of wound healing. In turn, dysregulation of the tissue homeostasis may lead to an irreversible fibrotic response, especially if the tissue injury is either severe or repetitive. Fibrosis is a hallmark of numerous systemic autoimmune disorders, such as rheumatoid arthritis, scleroderma, and progressive systemic sclerosis [5]. In cancer patients, fibrosis may follow chemo- and radiotherapy [6,7].

Presently, more than a dozen animal models, such as genetically modified mice and mice with chemically induced fibrosis, are available. These experimental models are used to explore the histological features of the scarred tissue and the associated changes in the expression of disease-relevant genes (rev. in [8]). Although some aspects of fibrosis in humans and mice differ, the animal models would help us explore the early stages of this pathological condition. Due to slow, almost asymptomatic initial onset, they frequently remain undiscovered during the evaluation of human patients. Furthermore, the animal models also would let us assess the role of specific genes in the pathogenesis of human fibrosis. In addition, they would allow us to evaluate new experimental therapies.

Obtaining the experimental model of bleomycin-induced skin fibrosis requires multiple consequent injections of mice skin with bleomycin [9,10]. Bleomycin is an antibiotic with a robust antiproliferative effect. In mammalian cells, bleomycin interferes with the inclusion of thymidine deoxyribonucleoside triphosphate into the DNA and causes the appearance of DNA breaks. To establish the named model, the experimenters inject bleomycin regularly until they observe the desired pathological changes, such as epidermal hypertrophy and dermal fibrosis, consisting of an accumulation of collagen and dense extracellular matrix material, and adipose atrophy becomes evident. Then, there is a need to wait until the inflammatory response subsides and fibrosis occurs to perform the necessary experimental procedures. Upon completion of the experimental protocol, the experimenters evaluate the expected therapeutic effects and analyze the role of selected genes in the pathological process using histological methods and data on gene expression in the collected samples.

Along with transforming growth factor-β (TGF-β) and decorin (DCN), matrix metalloproteinases (MMPs) are the most characteristic biomarkers of cutaneous fibrosis. In the affected area, MMPs maintain the balance between the accumulation of the extracellular matrix (ECM) and its breakdown [2]. TGF-β activates the biosynthesis of collagen and inhibits the degradation of ECM proteins. Furthermore, it stimulates the migration of macrophages and fibroblasts to the wound. At the site of damage, TGF-β promotes the interactions of cells with the ECM and the differentiation of myofibroblasts to fibroblasts [11]. It also downregulates the biosynthesis of collagen. DCN downregulates collagen fibrillogenesis via the inactivation of TGF-β. In turn, a suppression of DCN distorts the structure and organization of collagen fibers [12]. MMPs are responsible for changes in the composition of the ECM. Being directly involved in the pathogenesis of fibrosis, they contribute to skin remodeling and re-epithelization. MMPs secreted by immune cells facilitate their migration to sites of inflammation to degrade damaged cells and malfunctioned biomolecules. The recovery of scarred tissue is also accompanied by significant changes in the activity of proteolytic enzymes, including MMPs [13,14]. These make MMPs valuable biomarkers of cutaneous fibrosis and prove their relevance for experimental studies and clinical applications.

The previous experimental works performed on animal models suggest that modulating active MMPs can be beneficial for patients with skin fibrosis to restore normal tissue homeostasis [15]. In this regard, careful and cautious management of MMPs in disease-affected tissues and organs will help to suppress the inflammatory response and downregulate the infiltration of immune cells [16]. For this reason, manipulation of MMPs requires an understanding of their role at various stages of the disease, including the early stages when it may not be easy to diagnose. This paper aimed to evaluate changes in the expression of MMP genes: *Mmp2*, *Mmp3*, and *Mmp9* caused by laser therapy in the experimental model of bleomycin-induced skin fibrosis.

## 2. Materials and Methods

### 2.1. Lab Animals

Mice were purchased from the Stolbovaya Research Center for Biomedical Technologies at the Federal Medical and Biological Agency of Russia (Stolbovaya, Moscow region, Russia). The study involved 21 female Balb/c mice (7–8 w. o.). The weight of the mice ranged from 20 to 30 g. Mice were housed in an air-conditioned room at a temperature of 21–23 °C on 14 h light cycles since day 1, with 4–5 animals per cage. The air humidity was between 50 and 65%. Mice received a balanced diet (Laboratorkorm, Moscow, Russia). They also had unlimited access to drinking water. The quarantine lasted for two weeks. Before the experiment, the mice were anesthetized with a combination of ketamine and xylazine (80–100 and 7.5–16 mg/kg, respectively) delivered intraperitoneally in 100 μL. Before sampling the skin, the animals received a lethal dose of sodium pentobarbital (500 mg/kg) administered through the tail vein.

For the experiment, the animals were divided into three groups (7 mice per group) Animals were ranked by weight and distributed into three treatment groups after randomly settling the first animal. The breeders preselected the mice to exclude littermates from the same shipment. We did not perform a paternity test on the mice in the lab. The animals were injected with either saline or bleomycin intradermally by a staff veterinarian who had not been informed of the following treatment options. The first group of animals received injections of 100 µL of saline (0.9% sodium chloride) intradermally. The second and third groups of mice received injections of 0.1 U bleomycin in 100 µL of saline. The injections continued every other day for 19 days in the interscapular region of the back. On the 30th day of the experiment, animals that belonged to the third group were irradiated with a SmartXide DOT^®^ CO_2_ laser (DEKA, Manchester, NH, USA). The dose of irradiation was 18.2 J/cm^2^. The laser was preset to discrete mode. Samples were collected 48 h after irradiation by a lab technician who was unaware of the treatments received by the animals and the principles of their grouping. Specimens of healthy skin assigned to the correlation analysis were collected 2 cm away from the site of damage. Skin samples assigned to real-time PCR were rinsed in ice-cold phosphate-buffered saline (PBS) to remove excess blood, frozen in liquid nitrogen, and stored at −96 °C until needed for experiments. Samples designated for histology were fixed in 10% formalin and embedded in paraffin. Paraffin blocks were sectioned at eight μm thickness and stained with hematoxylin–eosin (H + E) following a standard protocol by a lab member unaware of the experimental design and used treatment options. Briefly, the prepared sections were incubated at 37 °C for 30 min and deparaffinized by consequent washing in xylene (2 times, 30 min each) and 100% ethanol (2 times, 10 min each). Deparaffinized sections were hydrated by washing in 90% ethanol for 5 min and then in 70, 50, and 30% ethanol (1 min each). After rinsing in PBS for 5 min, sections were stained with hematoxylin for 5 min and eosin for 30 s and washed with tap water after each incubation. After removing the excess water, sections were dehydrated with 2 changes of xylene (10 min each), mounted in VectaMount^®^ Express mounting medium (Vector Laboratories, Newark, CA, USA), coverslipped, and sealed with transparent nail polish. After staining, the sections were examined using bright field microscopy with an AxioVert.A1 microscope (Zeiss, Oberkochen, Germany). The thickness of the epidermis and the total thickness of the skin were measured using ImageJ freeware (NIH, Bethesda, DE, USA) by a lab member unaware of treatments received by animals and the principles of their grouping.

### 2.2. Purification of Total RNA

Total RNA was isolated using the TRIZOL reagent (Thermo Fisher Scientific, Waltham, MS, USA) according to the protocol provided by the manufacturer. Briefly, the tissue samples were homogenized in TRIZOL. Then, they were subjected to extraction with chloroform (5:1). After centrifugation (12,000× *g*, 15 min, 12 °C), the upper (aqueous) phase was precipitated with isopropanol (1:1). The pellet was washed in 70% ethanol, spun down (8000× *g*, 5 min, 8 °C), and dissolved in nuclease-free deionized water (Evrogen, Moscow, Russia). The isolated total RNA was treated with RNase-free DNase I (Qiagen, Hilden, Germany) to remove the traces of genomic DNA. The concentration of RNA was assessed spectrophotometrically with Nanodrop (Thermo Fisher Scientific) equipment according to the instructions provided by the manufacturer. If the A260/A280 ratio in one of the samples did not exceed 2.0, the samples were re-purified with Clean RNA standard kit (Evrogen). The quality of the preparations was verified by electrophoresis in 1.5% agarose gel in non-denaturing conditions.

### 2.3. Real-Time PCR

Before the experiment, total RNA was converted to complementary DNA (cDNA) using the MMLV RT reagent kit (Evrogen) according to the manufacturer’s protocol. The amplification of DNA was performed on a real-time DT prime 5M1 instrument (DNA-Technology, Moscow, Russia) using an SYBR Green master mix supplied by Evrogen, according to the manufacturer’s recommendations. The primers (Table 1) were designed using NCBI Primer BLAST [17] and checked with a multiple primer analyzer (Thermo Fisher Scientific) for the formation of potential secondary structures, such as loops and dimers, purchased from DNA-Synthes (Moscow, Russia). The following conditions were used to amplify the DNA: 3 min at 95 °C, followed by 40 cycles of incubations at 94 °C for 30 s and 57 °C for 15 s. Each sample served as input to prepare three sets of probes that would be sufficient to repeat the experiment three times. The results were analyzed using the standard 2^−ΔΔCt^ method [18] to compare the levels of expressed genes. Each ΔCt value was calculated as ΔCt = Ct (tested gene) − Ct (housekeeping gene). ΔΔCt was calculated as ΔΔCt = ΔCt (treated skin) − ΔCt (healthy control). The experiments were repeated three times for each sample by a lab member unaware of the experimental design. The raw data were normalized to the expression level of the housekeeping gene (18S RNA) and processed using a built-in computer program distributed by the manufacturers.

### 2.4. Statistical Analysis

The results were presented as mean value ± standard error (m ± SE). The comparison of two independent means was performed using Prism software (version 5.01; GraphPad Software Inc. San Diego, CA, USA) with the significance of the difference between the groups assessed by a one-way ANOVA followed by Tukey’s post hoc comparison of means test. The differences between the means were considered significant if the probability of accepting the null hypothesis did not exceed 0.05. The sample size was calculated using the “resource equation method” [19].

## 3. Results

### 3.1. Laser Therapy Normalizes the Histological Features of Skin Damaged with Bleomycin

**Figure 1:**
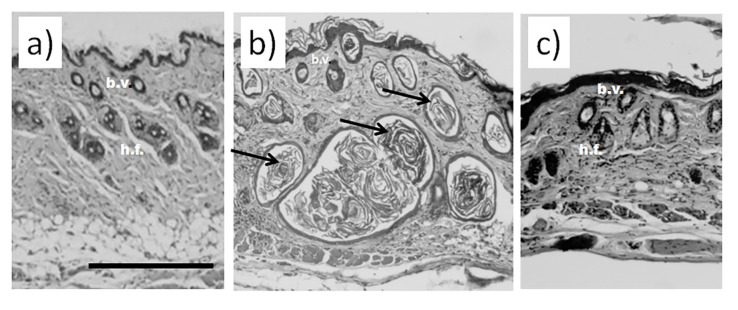
Histological analysis of murine skin. (**a**) Skin of an animal injected with saline without visible damage; (**b**) murine skin after injections with bleomycin; (**c**) murine skin after the course of bleomycin and subsequent laser therapy. Scale bar is 100 μm. Bundles of collagen fibers in bleomycin-treated mice are indicated by black arrows b.v.—blood vessels; h.f.—hair follicles.

**Figure 2:**
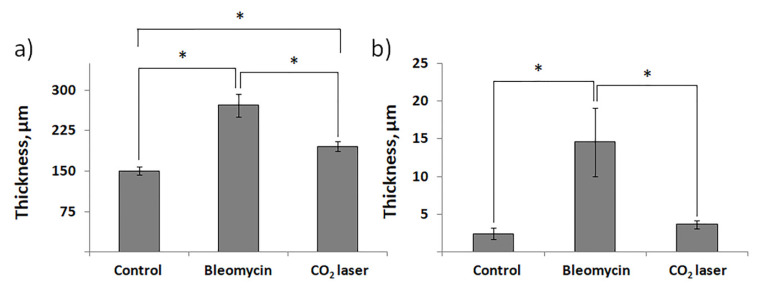
The thickness of murine skin before and after injections with bleomycin and the following laser therapy. (**a**) The total thickness of the skin; (**b**) thickness of the epidermis. “Control”—mice injected with saline; “Bleomycin”—mice injected with bleomycin; “CO_2_ laser”—mice injected with bleomycin after irradiation with a CO_2_ laser. Statistically significant differences of the means in groups less than 0.05 are indicated by asterisks (*).

Histological examination did not reveal any signs of inflammation or fibrosis in the skin of the animals treated with saline (Figure 1a). Contrarily, we observed significant thickening and swollenness in the skin of bleomycin-treated mice (Figure 1b). Compared to the animals injected with saline, the skin of the animals injected with bleomycin exhibited a significant structural remodeling of the dermis. The dermis contained multiple bundles of collagen fibers traceable throughout the upper and lower dermis. Their presence reflected an increased deposition of collagen (Figure 1b). Subcutaneous fat was reduced and partially replaced by connective tissue. In the upper dermis, the blood vessels were dilated and characterized by pronounced thickening of the walls. In some of them, the endothelial cells distinctly bulged toward the lumen. Many of the blood vessels contained a fair number of immune (nucleated) cells. In addition, we observed almost complete atrophy of the skin appendages, such as hair follicles. The total thickness of the skin increased twofold (Figure 2a). We also saw evident signs of epidermal hyperplasia (Figure 2b). At the same time, we did not observe morphologically visible epidermal damage following laser irradiation. However, the therapy partially normalized the histological features of the skin. 

The subsequent treatment of mice with laser therapy significantly normalized the histological features of the affected skin (Figure 1c). In mice subjected to laser therapy (group 3), we noticed fever fibrotic foci compared to the bleomycin-only control (group 2). The diameters of blood vessels reduced. The skin thickness also decreased (Figure 2a). We also noticed a faster reappearance of hair follicles compared to group 2. In addition, the absence of significant differences in the epidermal thicknesses after laser therapy and healthy skin (group 1) suggests a cessation of hyperplasia (Figure 2b). 

### 3.2. Analysis of Gene Expression Revealed Significant Changes in the Expression of Matrix Metalloproteinases

**Figure 3:**
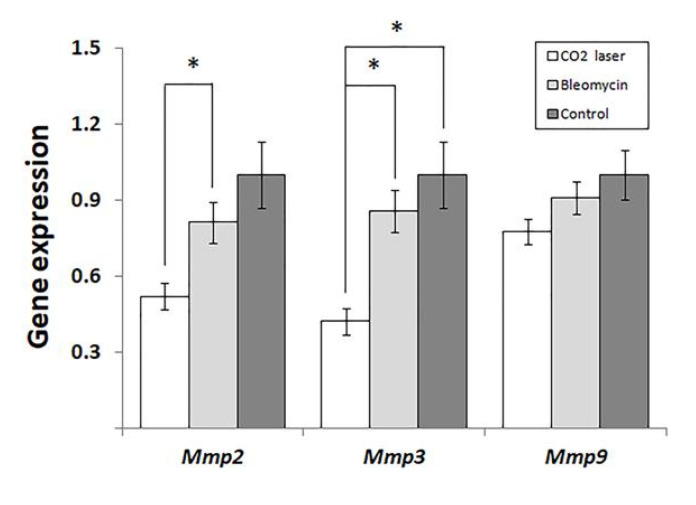
Changes in the expression of matrix metalloproteinases in mice injected with saline and bleomycin-injected mice before and after the laser therapy. “Control”—mice injected with saline (group 1); “Bleomycin”—mice injected with bleomycin (group 2); “CO_2_ laser”—mice injected with bleomycin after the laser therapy. Statistically significant differences of the means in groups less than 0.05 are indicated by asterisks (*).

Analyzing the expression of MMPs, we found that laser therapy of mice injected with bleomycin (group 3) significantly downregulated *Mmp2* and -*3*. (Figure 3) compared to sham and negative control (groups 1 and 2, respectively). We also discovered a similar trend in the expression of *Mmp9* (Figure 3). However, the observed changes were not statistically significant (*p* = 0.065). 

**Figure 4:**
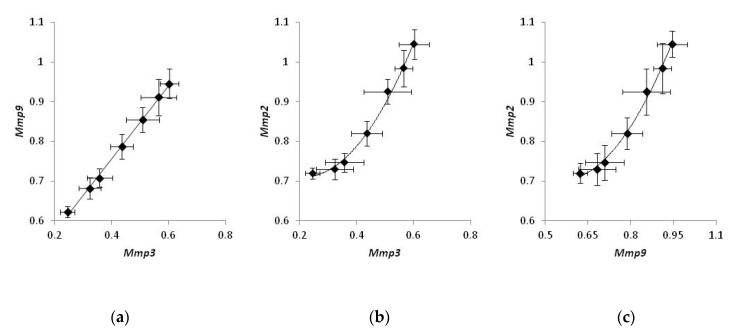
Coordinated gene expression of matrix metalloproteinases in bleomycin-injected mice treated with laser therapy. Samples of non-irradiated healthy skin taken from the same animals were used as control. Correlations in the expression of *Mmp3* and *Mmp9* (**a**), *Mmp3* and *Mmp2* (**b**), *Mmp9* and *Mmp2* (**c**).

Comparing the expression of MMPs in individual mice treated with a CO_2_ laser, we noticed that the expression levels of *Mmp2*, *Mmp3*, and *Mmp9* were strongly correlated. The levels of *Mmp3* were proportional to the levels of *Mmp9* (Figure 4a), and the experimental data could be fit with a linear regression (*r*^2^ = 0.97). On the other hand, the correlation between *Mmp2* and either *Mmp3* or *Mmp9* (Figure 4b,c) was non-linear and could be approximated with a polynomial function. (*r*^2^ = 0.98 and 0.96 and, respectively). At the same time, a similar analysis of mice belonging to two other groups did not exhibit a strong correlation in the expression of any pair of MMPs. 

## 4. Discussion

In this study, we examined the skin samples of animals (group 3) treated with the SmartXide DOT^®^ CO_2_ laser (DEKA) and animals of two control groups. Animals of group 1 (sham control) received injections of saline instead of bleomycin, and these mice did not receive the following treatment with laser therapy. Animals of group 2 (negative control) received injections of bleomycin. However, they did not receive laser therapy. We also analyzed changes in the expression of MMPs, namely *Mmp2*, *Mmp3*, and *Mmp9*, in all three groups of mice. 

The results of the histological analysis revealed that we successfully established the experimental model of bleomycin-induced skin fibrosis. In this regard, we found that the skin of animals injected with bleomycin was significantly thicker than that of the sham control animals (see groups 1 and 2 in Figure 1). Moreover, the skin of bleomycin-induced mice exhibited evident signs of epidermal hyperplasia (Figure 2a,b). The dermis contained multiple bundles of misarranged collagen fibers (Figure 1b). Subcutaneous fat partially replaced the connective tissue. In addition, the skin lost most of the appendages.

We also showed the skin remodeling effect of fractional lasers through the histological study of the collected skin samples. We report that, after laser therapy, the thickness of the skin became comparable to that in the sham control (Figure 2a). Similar thicknesses of the epidermis between groups 1 and 3 indicated cessation of hyperplasia (Figure 2b). The irradiated dermis (group 3) also contained fewer fibrotic foci than the dermis of non-irradiated bleomycin-treated mice (group 2). 

The analysis of gene expression (Figure 3) indicated that laser therapy of bleomycin-injected animals (group 3) significantly reduced the expression of *Mmp2* and -*3* compared to the other groups of mice. The expression levels of MMPs in healthy skin are low according to [20]. After an injury, several MMPs, including *Mmp2*, *Mmp3*, and *Mmp9,* become upregulated [21]. In human patients, the level of MMP2 is significantly higher in collagen bundle regions of keloids compared to non-collagen bundle regions [22].

Earlier, Matuszczak et al. [23] reported that therapy with an ablative fractional CO_2_ laser decreased the plasma level of MMP2 and α1- type I collagen in human patients with hypertrophic burn scars. At the same time, the therapy improved the texture, color, and functioning of the affected areas of the skin. In addition, the authors did not observe any recurrence or worsening of scar appearance up to 4 years after the treatment.

An obvious way to improve the results of laser therapy is to combine skin irradiation with a supplementary treatment. In this regard, Lee et al. [24] discovered that applying adipocyte-derived stem cell-containing medium supplemented with niacinamide, the precursor of the cofactors niacinamide adenine dinucleotide (NAD) and its phosphate derivatives (NADP), after irradiation of skin with an ablative fractional laser (AFL) improved skin texture and pigmentation. They also significantly suppressed the expression of *MMP1*, *MMP2*, and proinflammatory cytokines *IL1α*, *-1β*, and *-6 i*n the following in vitro experiments.

Using fractional lasers for scars has several advantages (rev. in [25]). Heating the dermis to 50–70 °C, irradiation with a laser produces ~3 mm deep microscopic skin lesions, initiating rapid wound healing in the affected area. Inducing collagenases reverses the accumulation of collagen fibers in the dermis. Furthermore, fractional lasers induce conformational changes in the collagen structure and the formation of new collagen fibers due to the produced thermal effect. Specifically, non-ablative fractional lasers (NAFLs) stimulate dermal remodeling and improve the functionality of the epidermal barrier. In addition, applying the therapy during the early phase of wound healing shrinks the microcapillaries, reducing the blood flow. These advantages are crucial, especially for burn scars that often penetrate the dermis. At the same time, using an AFL with incorrect settings may worsen scarring [26]. It can also cause postinflammatory hyperpigmentation (PIH) [27].

In a recent paper, Chung et al. [25] reported that the AFL and NAFL treatment of pigs significantly changed the expression of Mmp2 and Mmp9. In ELISA experiments, the authors showed that the level of Mmp2 significantly increased after low- and high-energy NAFL treatment compared to the sham control and AFL treatment. In turn, the differences between the sham control and AFL were insignificant. In qPCR experiments, Chung et al. [25] did not see significant differences between groups of hypertrophic scars subjected to AFL and NAFL treatment. Contrarily, the expression of *Mmp2* in ALF-treated mice significantly exceeded that in the sham control. In this regard, we can propose that the observed changes between the two groups of scars treated with an NAFL likely reflected a progressive increase in *Mmp2* expression.

In our study (Figure 3), we show significant suppression of *Mmp2* at the early stages of post-therapeutic recovery. Accordingly, the following increase in the expression of *Mmp2* would explain the absence of statistical differences in Chung’s qPCR experiments [25] and the accumulation of secreted *Mmp2* detected by ELISA. The same proposal can explain higher levels of *Mmp2* in animals treated with low- and high-energy NAFLs.

In addition, we would make a short comment on the unconventional experimental design used in Chung’s study that could influence their final results. The authors produced 40 hypertrophic burn scars on the abdomens of two animals. After epithelialization, they randomly subdivided scars into four groups and applied different treatments to each group. In this regard, we have to mention that it would be unusual to apply the same practice, i.e., simultaneously using a few treatments on the same human patient. Otherwise, controversial outbound signals released by inflamed tissue could originate the wrong type of immune response and cause an unexpected result.

Forty-eight hours after the therapy, the expression of *Mmp3* significantly decreases, similar to *Mmp2* (Figure 3). As found earlier, stromelysin 1, encoded by *Mmp3*, contributes to the degradation of collagens, elastin, laminins, and other proteins of the ECM [28]. Furthermore, stromelysin 1 activates several matrix metalloproteinases: interstitial collagenase/MMP1, matrilysin/MMP7, and gelatinase B/MMP9. These enzymes are necessary for the degradation of proteins of the dermal ECM [29]. In cutaneous fibrosis, MMP3 is responsible for the contraction of fibroblasts and initiation of wound contraction [30].

Our data are in agreement with similar results obtained by others. For instance, Amann et al. reported that treatment of human three-dimensional (3D) organotypic skin models with a fractional erbium glass laser decreased the expression of MMP3 for five days after the therapy [31].

In our experiments, the expression of *Mmp9* follows the same trend as two other MMPs (Figure 3). However, the changes between groups 2 and 3 are not statistically significant (*p* = 0.065). The data obtained by others [13] suggest that the expression level of *Mmp9* after laser therapy reaches the baseline faster than that of Mmp3. For instance, the expression of MMP9 increased on day 5 in Amman’s qPCR experiments, although the authors did not find significant differences from the control [31]. 

Exploring the correlations of the expression levels, we found that the expression of MMPs in bleomycin-injected mice is well-coordinated. The levels of *Mmp2*, *Mmp3*, and *Mmp9* were strongly correlated. We also noticed that changes in the expression level of *Mmp3* were proportional to those of *Mmp9,* and the corresponding data points could be fit with a linear regression (Figure 4a). Contrarily, the correlation between *Mmp2* and either *Mmp3* or *Mmp9* was non-linear and could be better approximated with a polynomial regression (Figure 4b and c, respectively). Non-linear correlations in gene expression of *Mmp2* and two other MMPs may suggest that the biosynthesis of *Mmp2* mRNA can be controlled by an enhancer. 

According to the published data [32], the proposed enhancer resides ~1500 base pairs upstream of the coding area. Its sequence contains a binding site for the transcription factor AP1, and its activation occurs during a traumatic injury of the tissue due to the binding to the enhancer of either Fosl1−JunB or FosB−JunB heterodimers of AP1 [33]. As we hypothesize, activation of the enhancer could produce an additional positive effect on the transcription of *Mmp2* and accelerate the biosynthesis of *Mmp2* mRNA at the site of damage compared to *Mmp3* and *Mmp9* mRNAs. According to another report, the enhancer turns to the active state following demethylation. During fibrogenesis, the enhancer becomes hypomethylated [34], and it increases the expression of *Mmp2*. Accordingly, transcriptional repression of the enhancer should produce a robust suppressive effect on the transcription of the *Mmp2* gene compared to two other mRNAs in our experiments (Figure 4). At the same time, the absence of similar strong correlations within groups 1 and 2 suggest that, in mice not subjected to the laser therapy, the expression levels of MMPs remained close to their physiological values.

To date, there is insufficient information on the molecular mechanism that regulates the expression of MMPs in laser-irradiated skin. According to Li et al. [35], the irradiation of the skin with a laser causes significant rearrangements in the 3D architecture of chromatin that produce changes in gene expression. Some of these changes occur due to the upregulation of IL-1β and the subsequent activation of the NFκB signaling pathways [36]. The others influence the availability of distant enhancers [37]. Summarizing the data cited in this paper, we presume that the regulatory mechanism controlling the expression of MMPs by laser irradiation is tissue-specific and scale-dependent since different devices and experimental conditions are likely to produce various outcomes.

We have to admit that our study has limitations. First, we performed it in a mouse model, and, as we mentioned above, the wound-healing process in mice and humans is not the same. They are likely caused by differences in the immune systems and wound contraction mechanisms (rev. in [38]). Furthermore, mouse skin is much thinner and loosely attached to the underlying fascial tissue. Consequently, differences in skin tension cause different physiological responses to wounding. Third, human and murine skins differ in their density and size of hair follicles. The latter cannot be ignored because hair follicles contain stem cells that may contribute to wound healing. In addition, mice skin does not contain sweat glands, except on foot pads. On the other hand, we show that the experimental model of bleomycin-induced skin fibrosis could be used to explore the beneficial effects of laser therapy. Similarly to other animal models, this model can be used to perform as gene (protein) regulation as well as histological analysis. 

## 5. Conclusions

In conclusion, our histological analysis revealed that laser therapy partially normalized the structure of fibrotic murine skin in the experimental model of bleomycin-induced skin fibrosis. We also found significant changes in the expression of *Mmp2* and -*3* and statistically insignificant changes of *Mmp9* (*p* = 0.065). In this regard, the obtained results demonstrated the efficiency of laser therapy and confirmed the efficiency of MMPs as biomarkers of skin fibrosis. In the paper, we speculate that the *Mmp2*, *-3*, and *-9* can be responsible for the acceleration of the inflammatory response and the following progression of skin fibrosis. In addition, the named MMPs, especially *Mmp2*, can be used to monitor the course of skin damage. At the same time, controlling the expression of MMPs relevant for skin fibrosis is important because of the participation of MMPs in restorative and pathological repair [39], suppression of the inflammatory response and the infiltration of immune cells [16]. For instance, it will help to optimize the degradation of collagen and stimulate the biosynthesis of elastin. At the same time, managing the expression of MMPs and their activation in fibrotic skin requires a better understanding of their role in this pathological condition. 

## Figures and Tables

**Table 1:** Gene-specific primers used in the qPCR experiments.

Gene	Reference Sequence	Primer Name	Primer Sequence	Product Size, bp
*MMP2*	NM_008610.3	MMP2 forward	CCCTGATAACCTGGATGCCG	214
		MMP2 reverse	AGGCTGCTTCACATCCTTCAC	
*MMP3*	NM_010809.2	MMP3 forward	CATGAACTTGGCCACTCCCT	179
		MMP3 reverse	GTGGGTACCACGAGGACATC	
*MMP9*	NM_013599.5	MMP9 forward	TGGTCTTCCCCAAAGACCTG	216
		MMP9 reverse	CACAGCGTGGTGTTCGAATG	
*18S RNA*	NR_003278.3	18S RNA forward	GCAATTATTCCCCATGAACG	123
		18S RNA reverse	TTGGATGGTTTAGTGAGGCC	

## Data Availability

Not applicable.
